# Vaccine-Induced Thrombotic Thrombocytopenia: A Case of Splanchnic Veins Thrombosis

**DOI:** 10.7759/cureus.23507

**Published:** 2022-03-26

**Authors:** Safwan Abbasi, Anas Alsermani, Abdulaziz Alsegayyir, Talal Altahan, Maamoun Alsermani, Sami Almustanyir

**Affiliations:** 1 College of Medicine, Alfaisal University, Riyadh, SAU; 2 Internal Medicine, Prince Mohammed bin Abdulaziz Hospital, Riyadh, SAU; 3 Clinical Affairs, Prince Mohammed bin Abdulaziz Hospital, Riyadh, SAU; 4 Internal Medicine, Ministry of Health, Riyadh, SAU

**Keywords:** thrombosis, heparin induced thrombocytopenia (hit), platelet -factor 4, covid-19 vaccine complication, covid 19, vaccine-induced thrombotic thrombocytopenia (vitt)

## Abstract

Vaccines have been vital in preventing and curbing the spread of SARS-CoV-2 infection. Adenoviral vector-based vaccines, namely the ChAdOx1-S vaccine (AstraZeneca, Cambridge, UK) and Ad26.COV2.S (Janssen; Johnson & Johnson, New Brunswick, NJ, USA), have been associated with a possibly fatal adverse event known as vaccine-induced thrombotic thrombocytopenia (VITT), wherein thrombi form in unusual sites, mainly the cerebral and splanchnic veins. With the female gender predominantly affected, patients present with headache, abdominal pain, neurological symptoms and fever. It is hypothesized that certain components of the vaccine, including the adenovirus vector, may trigger the formation of antibodies against platelet factor 4 (PF4). The antigen-antibody complexes that form thereafter then activate a cascade of reactions eventually leading to the consumptive coagulopathy. This pathogenesis closely resembles a well-understood complication of heparin, known as heparin-induced thrombocytopenia. The lab results in VITT are reflective of its proposed pathophysiology: low platelets, low fibrinogen and high D-dimer, in addition to elevated anti-PF4 titers are classic findings. Treatment mainly includes non-heparin anticoagulants, intravenous immune globulin (IVIG) and plasma exchange. There is some role for surgical intervention, such as mechanical thrombectomy. Mortality due to VITT is usually caused by cerebral hemorrhage. We describe a case of a 36-year-old female who presented with epigastric pain two weeks after receiving her first dose of the AstraZeneca vaccine, and upon workup, was subsequently found to have thrombosis of her right portal and right common iliac vein.

## Introduction

Vaccines have played a pivotal role in preventing and curbing the spread of the severe acute respiratory syndrome coronavirus 2 (SARS-CoV-2) infection. Since the start of the pandemic, numerous vaccines have emerged with varying efficacies, with most of them being generally safe. However, in a limited number of individuals, a potentially fatal adverse effect, termed as vaccine-induced thrombotic thrombocytopenia (VITT), has been noticed in conjunction with certain vaccines.

In February 2021, news of otherwise healthy recipients of the adenoviral vector-based ChAdOx1-S vaccine (AstraZeneca, Cambridge, UK) developing complications of thrombocytopenia and thrombosis in atypical locations (such as cerebral and splanchnic veins) were reported. Approximately two months later, similar descriptions of such complications were reported among the recipients of another adenoviral vector-based vaccine, Ad26.COV2.S (Janssen; Johnson & Johnson, New Brunswick, NJ, USA) [1-3].

Cerebral venous sinuses are most commonly involved, with cerebral venous thrombosis (CVT) occurring in 38-80% of the reported cases. This is followed by splanchnic veins. With the inclination of VITT causing thromboses of cerebral and splanchnic veins, patients typically present with neurological symptoms, headaches, nausea, vomiting and abdominal pain. Lab studies point towards a consumptive coagulopathy (elevated D-dimer, low platelets and fibrinogen), with the addition of anti-platelet factor 4 (PF4) antibodies detection [1,4]. Case fatality is reported to be up to 20%, with the predominant culprit being cerebral hemorrhage [1-3,5].

## Case presentation

A 36-year-old female teacher presented to the emergency room (ER) with epigastric pain that started a week ago. The pain was intermittent and described as being 8 out 10 on a severity scale, with food being the aggravating factor. She had received the ChAdOx1-S vaccine (AstraZeneca) two weeks prior to the onset of the pain. She is married with two children and had her last delivery six years ago. The rest of her obstetrics and gynecological history was unremarkable. Her family history was significant for deep venous thromboses (DVTs) in her sisters, although on further questioning, those episodes seemed to have been provoked. She does not have diabetes mellitus, hypertension or other chronic illnesses. She does not have personal or family history of any malignancies or hematological diseases, and does not smoke or drink alcohol.

She was worked up in a private hospital and found to have portal vein thrombosis on imaging, after which, she was referred to Prince Mohammad bin Abdulaziz Hospital in Riyadh, Saudi Arabia.

On admission, the patient was conscious, alert and oriented to time, place and person. Her vitals were stable and she was not in any active pain or distress. An abdominal exam revealed a soft and lax one with no tenderness. There was no lymphadenopathy or lower limb edema. The remainder of the physical exam was unremarkable.

Complete blood count (CBC) and peripheral blood smear performed were unremarkable, except for a platelet level of 155 x 10^9^/L, which was relatively low compared to her baseline of 350 x 10^9^/L. The coagulation profile revealed a normal prothrombin time, normal activated thromboplastin time, low fibrinogen and an elevated D-dimer of 15.95 µg/ml (reference range: 0-0.5 µg/ml FEU). Serum chemistry (electrolytes, blood glucose, albumin, AST, bilirubin, lipase, amylase, lipid profile) yielded normal results except for an elevated aspartate transaminase (AST) of 56 U/L (reference range: 10-45 U/L). Tests for inflammatory markers and autoimmune antibodies revealed a high C-Reactive protein, and normal levels of anticardiolipin and beta-2 glycoprotein antibodies. Her inherited thrombophilia screen (e.g., factor V Leiden, protein C/S deficiency, etc.) was also negative. Due to unavailability, anti-PF4 assays could not be performed. COVID-19 polymerase chain reaction (PCR) test was negative.

**Figure 1:**
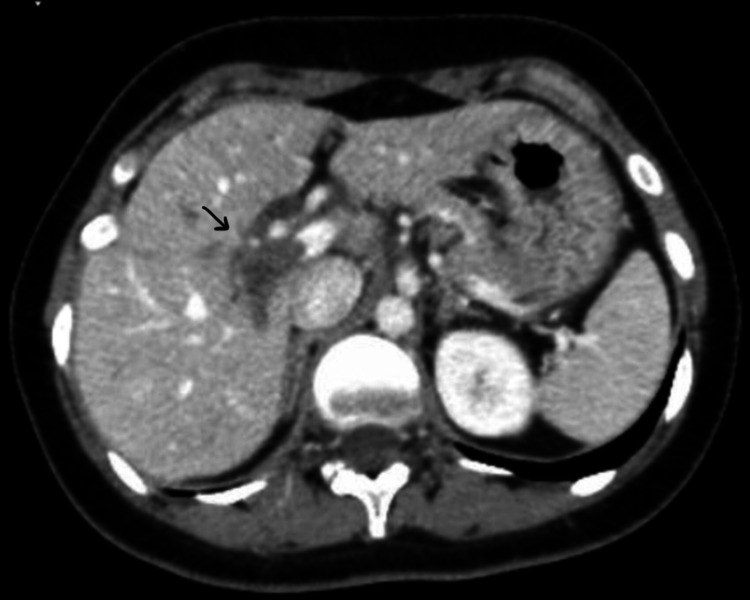
Contrast CT Abdomen (Axial Section)

A contrast-enhanced computed tomography (CT) scan of the abdomen was subsequently obtained which was significant for a thrombosed right portal vein at its branches in the right lobe of the liver (see Figure 1), and a partial thrombus noted at the proximal part of the right common iliac vein extending to the right internal iliac vein. The left and main portal veins, superior and inferior mesenteric veins, splenic veins, hepatic veins and inferior vena cava were all found to be patent. There were no areas of abnormal enhancement seen at the bowel loops and no signs of bowel obstructions were identified. Other than a prominent common bile duct, a minimal amount of free fluid in the pelvic cavity, a small fat-containing umbilical hernia, a right ovarian corpus luteum cyst, and a mildly thickened endometrium, no other abnormalities were found on the CT scan.

Suggestive lab results (low fibrinogen, high D-dimer and relatively low platelets), the presence of thrombosis in an unusual site, along with the fact that the patient was healthy with no previous history of thrombosis or provoking factors for it, except for the COVID-19 vaccine administration only two weeks back, raised the suspicion for VITT.

She was administered Apixaban as anticoagulation and intravenous immune globulin (IVIG) (1 g/kg for two days). Her platelets subsequently showed improvement (293 x 10^9^/L then 319 x 10^9^/L). For long-term management, she was given Apixaban for six months, and recanalization of the previously thrombosed veins was confirmed with CT scan five months after discharge.

## Discussion

Reports of thrombotic events following the administration of AstraZeneca (AZ) and Janssen; Johnson & Johnson (J&J) vaccines emerged in March 2021. The initial cases were reported by Schultz et al. from Norway when they described five patients admitted with thrombotic complications and thrombocytopenia following administration of the AZ vaccine [2]. Further reports of similar episodes in 11 patients were outlined by Greinacher et al. from Germany and Austria, and in 23 patients by Scully et al. from the UK [3,5]. While it seemed like the AZ vaccine was solely associated with such circumstances, comparable reports after the J&J vaccine came to light from the United States [6]. Owing to its thrombosis-related events and low platelets, this phenomenon came about to be known as vaccine-induced thrombotic thrombocytopenia (VITT).

The incidence of VITT in recipients of the responsible vaccines ranges from 1/20,000 to 1/100,000 [7]. Furthermore, VITT seems to affect relatively younger females. As of May 2021, out of the 309 cases reported in the UK, 169 were females (55%), and out of the 28 cases reported in the US, 22 were females (78%) [1]. The trend is similar in the initial reports from Germany, Austria, and Norway [2,3]. Regarding the most affected age group, the incidence of VITT is rare in those above 70 years. There is no agreeable correlation between VITT and concurrent presence of other medical diseases, usage of oral contraceptives, hormonal therapy, or even thrombophilia [1].

VITT has a predilection to cause thrombosis in cerebral venous sinuses, followed by splanchnic veins, particularly the portal vein, all of which are unusual sites [8]. According to the initial reports by Schultz et al., 80% of the cases had CVT. This figure was 90% in the German/Austrian report by Greinacher et al., 56.6% in the UK report by Scully et al., and 100% in the US report by Muir et al. [2,3,5-7]. Infrequently reported thrombotic events include pulmonary embolism, myocardial infarction, and middle cerebral artery stroke [5,8]. There also has been a single case of bilateral adrenal hemorrhage secondary to VITT [9].

When it comes to the clinical features of VITT, they are mainly dependent on the vessel wherein the thrombosis occurs. The most common symptoms are understandably severe headache or abdominal pain due to the involvement of cerebral veins or splanchnic veins respectively, but fever and abnormal neurological findings may also occur. In the case of a pulmonary embolism, patients may present with dyspnea, tachypnea, and tachycardia. In cases of the rarer sites of thromboses, chest pain, cardiovascular collapse or signs of limb ischemia may be the presenting complaints [1,7,8]. On average, patients present 5-24 days after being vaccinated, which in most cases, is their first dose [8].

The pathophysiology of VITT is not entirely understood, but most postulations point to it being an autoimmune phenomenon. The clinical features and lab results of VITT closely resemble that of heparin-induced thrombocytopenia (HIT), a hypercoagulable state that develops 4 to 10 days after exposure to heparin. The hallmark of HIT is antibodies formed against platelet factor 4 (PF4) complexed with heparin or large anions, for example, DNA or glycosaminoglycans (GAGs). These anti-PF4 antibodies can be detected by enzyme immunoassays and are found elevated in patients with HIT. The prothrombotic state is subsequently achieved when PF4-antiPF4 complexes crosslink Fc gamma RIIA receptors on platelets, monocytes, and neutrophils to trigger intracellular pathways that eventually bring about the hypercoagulable state [1,8]. An uncommon variant of HIT is autoimmune heparin-induced thrombocytopenia (aHIT), wherein anti-PF4 antibodies developed in patients not subjected to heparin [8].

The consumptive coagulopathy that develops thereafter in HIT or aHIT manifests classically with low platelet count, low fibrinogen, and high D-dimer levels [7]. Given the fact that comparable lab results, along with high anti-PF4 titers are also observed in VITT, this has indicated that the pathogenesis of VITT must be very similar to HIT. To be specific, the absence of heparin exposure means that VITT mimics aHIT more than HIT [1].

The question then arises is what triggered the formation of anti-PF4 antibodies in the first place. Since the vaccines with which VITT is associated are both adenoviral-vector based, it is hypothesized that the adenovirus vector itself precipitates the formation of the antibodies [7,8]. The counterargument to this is that adenovirus vector has been utilized in the manufacture of other vaccines such as Ebola and AIDS vaccines, albeit there have been no VITT-like adverse effects reported with either of them [8]. It may very well be that some other component(s) of the vaccine, such as polysorbate 80 may be the provoking factor in the formation of anti-PF4 antibodies although another source suggests that it is unlikely to play a role in the pathogenesis [8,10].

The management of VITT involves admission of the patient and treatment with non-heparin anticoagulants (such as argatroban) with or without intravenous immune globulin (IVIG). Platelet transfusions are to be avoided and in refractory cases, plasma exchange has also been employed in the management of patients. Surgical intervention has also been described and includes procedures such as mechanical thrombectomy and those to reduce the high intracranial pressure [1,7].

Cerebral hemorrhage superimposed on thrombosis occurs in 26% to 80% of individuals and is the major cause of death in patients with VITT [1].

## Conclusions

VITT may occur following vaccination with the adenoviral vector-based AstraZeneca and Janssen; Johnson & Johnson vaccines. Care providers are prompted to have a low threshold for detection of this potentially fatal complication and should be alarmed by headache, neurological signs, or abdominal pain presenting 5 to 24 days after the first dose administration. The workup of suspected cases includes a coagulation profile, D-dimer, fibrinogen, and anti-PF4 levels. Prompt diagnosis and treatment are vital to prevent morbidity and mortality.
